# Congenital arthrogryposis-hydranencephaly syndrome caused by Akabane virus in newborn calves of Basrah Governorate, Iraq

**DOI:** 10.14202/vetworld.2017.1143-1148

**Published:** 2017-09-29

**Authors:** K. M. Alsaad, H. H. N. Alautaish, M. A. Y. Alamery

**Affiliations:** Department of Internal and Preventive Medicine, College of Veterinary Medicine, University of Basrah, Basrah, Iraq

**Keywords:** Akabane virus, arthrogryposis-hydranencephaly, calves, Iraq

## Abstract

**Aim:**

The study was conducted in Basrah, Iraq, to diagnose congenital arthrogryposis-hydranencephaly syndrome caused by Akabane virus (AKAV) in calves.

**Materials and Methods:**

Affected animals (42 calves) are about 2-27 days old from both sexes show signs of arthrogryposis and hydranencephaly. Eight clinically healthy newborn calves were considered as controls. Diagnosis of AKAV was confirmed using a competition enzyme-linked immunosorbent assay test.

**Results:**

Results show that all affected calves were found seropositive. Furthermore, a significant increase in total leukocyte count in diseased calves due to a significant increase in the absolute lymphocyte number indicated in affected calves than in controls. Moreover, a significant increase in sedimentation rate of erythrocytes was also encountered in diseased calves than in controls. In addition, a significant increase in haptoglobin level and fibrinogen was also detected.

**Conclusion:**

Diagnosis of AKAV infection of Basrah Governorate, Iraq, will provide useful epidemiological information for cattle and other domesticated animals. Therefore, abortion could be prevented and controlled.

## Introduction

Akabane congenital arthrogryposis-hydranencephaly syndrome was an infectious disease of the bovine, ovine and caprine, and fetuses caused by intrauterine infection and interfere with fetal development after transmission to the pregnant dam by biting gnats or mosquito transmitted Akabane virus (AKAV) and some other antigenically related members of the Simbu group of arboviruses of the family Bunyaviridae [1,2]. The disease considered as the major cause of epizootics of congenital malformations in ruminants. Since the fetal infection may cause abortions, stillbirths, premature births, mummified fetuses, and various dysfunctions or deformities of fetuses or live born neonates [3,4].

The disease is common in numerous tropical and subtropical areas; they had been recognized in Asian countries, Australia, African countries, and some other areas of the world [5-7]. The virus is well known as a teratogenic type of pathogen and has the ability to cross through ruminant placenta during different stages of pregnancy period [2]. The stage of pregnancy in which the cow is infected determines the range and severity of clinical signs [8]. In calves born alive, the disease appears in different forms, calves infected at an older fetal age (5-6 months) are born with arthrogryposis which results from abnormal development of the spinal cord, thereby abnormal nerve supply of the musculature, resulting in failure of normal development of muscles; hence, ankylosis of the joints as a result of this failure may cause severe distortion. Furthermore, Oakley *et al.*, [9] added that calves which infected with early stages of pregnancy might get multiple limbs affected, while animals infected with later stages of pregnancy might only have a single affected limb. Moreover, earlier infection results in calves born of arthrogryposis and hydranencephaly were deficient of the cerebral cortex, and replacement of brain tissue by a fluid-filled sac is detected, and when calves affected at 3-4 months, fetal age hydranencephaly was primarily seen only [10].

The infection reported in cattle, sheep, goats, pigs, buffaloes, and camels, although antibodies were also detected in horses, no clinical evidence of fetal infection has been reported [11].

AKAV infection, which is a congenital arthrogryposis-hydranencephaly syndrome of bovine fetuses its sometimes also calling the curly calf disease, silly calves, or acorn calves [12,13]. The disease affected newborn animals as congenital anomalies. However, adult animals are not clinically affected while actively infected with the virus [3]. Moreover, the virus does not affect humans [2].

Spreading of AKAV was always related to the distribution of the insect vector populations and the seasonal conditions will approve their spread. Thereby, akabane disease may be seasonal and/or sporadic, especially in areas where the ectoparasites such as mosquito or midges are not endemic [14,15].

Reports of AKAV infection in Basrah Governorate, Iraq, is not existent and no information had been provided. Therefore, the present study was aimed at detecting the virus antibodies in diseased newborn calves with evaluation of acute phase response, erythrocyte sedimentation rate (ESR), total leukocyte count (TLC), and differential leukocyte count (DLC) in diseased calves of Basrah, Iraq.

## Materials and Methods

### Ethical approval

Ethical approval was not necessary for this study. However, samples were collected as per standard collection procedure.

### Animals

The study was conducted in Basrah Governorate, Iraq, and conducted on 42 calves (2-27 days old) from both sexes which show different clinical signs concerning arthrogryposis-hydranencephaly syndrome. Eight clinically healthy newborn calves were considered as controls. The diseased calves brought and clinically examined at the Consultant Veterinary Hospital belong to a College of Veterinary Medicine, University of Basrah, Iraq, during the period started from September 2014 to October 2016. The careful clinical examination was done for all animals. A complete animal history was obtained on presentation in the consultative clinic and emphasis was placed on clinical manifestations observed, course, and duration of the presenting complaint.

Indeed the authors can’t do the histopathology because is too hard and difficult, as all the diseased calves were still a life when it come to the clinic for the clinical examination and diagnosis. Moreover, it also too hard to convincing and For persuasion the calf’s owners to take biopsies before or autopsies for histopathological examination after slaughter their animals. Since some civilian rules are still hard dealing with less or un-educated farmers. The authors therefore, were confirmed there diagnosis only via the detection of the specific antibodies belong to the Akabane virus present in affected calves using, the Competition ELISA test.

In fact information about culicoides spp did not added to this study because the infection was occurred via intrauterine method as the affected calves are of (2-27) days old and found all clean from the external ecto-parasites infection.

### Samples

About 10 ml of blood were collected through jugular vein puncture. Ethylenediaminetetraacetic acid-mixed blood (2.5 ml) used to determine, TLC (Hematology analyzer, Genex, USA), DLC was estimated through Giemsa-stained blood smears, and ESR by the wintrobe tube method [16]. Other 2.5 ml of blood mixed with trisodium citrate (using plasma) to determine fibrinogen using commercial kits (Biolabo, France).

The authors assessed the Fibrinogen to evaluate the acute phase response related to the arthrogryposis – hydranencephaly syndrome, (Since Fibrinogen is considered as one of the acute phase protein). As, no previous studies have found the relationship or evaluate this response. Moreover, Fibrinogen was evaluated, as the syndrome affected joints (ankylosis and distortion of the joints) and failure of normal development of muscle tissues. Since, fibrinogen values might change, so the authors, want to find how the change will happen during this syndrome and its confirmed. Furthermore, the acute phase response can also evaluate the tissue damage result from infection. Moreover, the acute-phase response had a large number of physiologic, biochemical, and even nutritional changes involving different organs away from the site (s) of inflammation.

Serum was analyzed for haptoglobin using the enzyme-linked immunosorbent assay (ELISA) technique (Biotechnology Co., China) estimated according to manufacturer’s instructions. Commercial ELISA Kits (Akabane Competition, Lillidale Diagnostics Ltd., England) was used for detection of AKAV antibodies in serum samples of calves according to manufacturer’s instructions.

### Statistical analysis

Statistical analysis using Student’s t-test was performed, and the significance of variations in diseased and healthy animals was evaluated [17].

## Results

**Figure-1:**
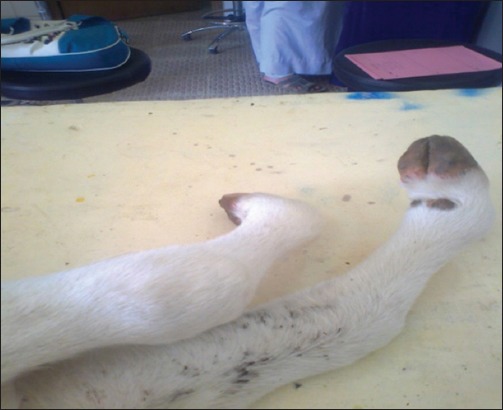
Arthrogryposis, animal shows curled toes with ankylosis.

**Figure-2:**
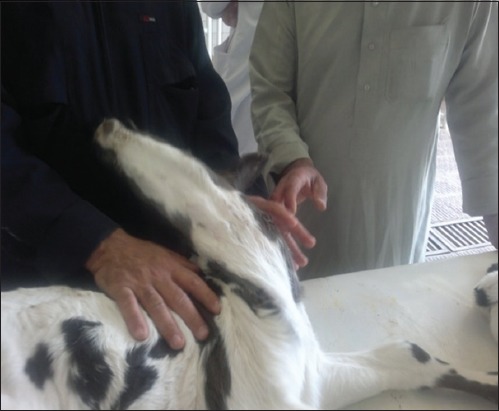
Hydranencephaly, animal shows torticollis of head and/or on neck.

Diseased calves show signs of dullness (52.3%), lateral recumbency (73.8%), with signs of arthrogryposis and hydranencephaly which include appearance blindness (26%), nystagmus (31%), deafness (16.6), torticollis of the head, and/or on the neck (54.7%) (Figure-1). Unable to suck the udder (Since the diseased animal have no ability to stand) (78.5), curled toes of fore and/or hind legs with ankylosis, as most joints are rigid and fixed in flexion (50%) (Figure-2). Tremor (19%) and in some cases, paralysis of hind limbs (14.2%) were detected (Table-1). Moreover, on clinical examinations, there was a significant increase (p<0.05) in body temperature, respiratory, and heart rate of diseased calves compared with controls (Table-2).

**Table-1:** Clinical signs showed by affected calves.

Clinical signs	Number of affected animals (%)
Dullness	22 (52.3)
Lateral recumbency	31 (73.8)
Appearance blindness	11 (26)
Nystagmus	13 (31)
Deafness	7 (16.6)
Curled toes of fore and/or hind legs with ankylosis	21 (50)
Unable to suck	33 (78.5)
Torticollis of the head and/or on the neck	23 (54.7)
Tremor	8 (19)
Paralysis of hind limbs	6 (14.2)

**Table-2:** Clinical examinations of affected and control calves.

Parameters	Controls n=8	Disease calves n=42
Body temperature/°C	38.8±0.63	40.2±1.53**
Respiratory rate/min	23.4±3.2	42.3±4.5**
Heart rate/min	112.3±4.22	162.6±7.34**

Values are mean±standard error of mean.

** p<0.05

Diagnosis of AKAV was confirmed using the competition ELISA, and the results show that all samples were found seropositive (Table-3).

**Table-3:** Detection of specific antibodies against AKAV using a competition ELISA.

Animal	Number of samples	Seropositive	Seroprevalence %	OD values/ELISA Mean±SE
Affected calves	42	42	100	0.021±0.008
Controls	8	0	0	0.0422±0.002

OD=Optical density, ELISA=Enzyme-linked immunosorbent assay, SE=Standard error, AKAV=Akabane virus

Data onto hematological parameters indicate a significant increase (p<0.05) in TLC in diseased calves due to a significant increase (p<0.05) in the absolute lymphocyte number indicated in infected calves than in controls. Moreover, a significant increase (p<0.05) in sedimentation rate of erythrocytes was also encountered in diseased calves than in controls (Table-4).

**Table-4:** Hematological parameters of affected and control calves.

Parameters	Controls n=8	Diseased calves n=42
TLC×10^3^	9.74±3.25	15.88±3.45**
Lymphocytes (absolute)	4233±46.22	9245.61±67.56**
Neutrophils (absolute)	5320±521.11	5498.43±54.18
Monocytes (absolute)	175±44.87	177±56.73
Eosinophiles (absolute)	222±71.54	217.45±30.83
Basophiles (absolute)	32±9.12	36.56±13.73
ESR mm/24 h	6.78±5.28	32.27±8.64**

Values are mean±standard error of mean.

** p<0.05.

TLC=Total leukocyte count, ESR=Erythrocyte sedimentation rate

Results are also to show a significant difference in acute phase response. Since these results revealed a significant increase (p<0.05) in haptoglobin level and fibrinogen statistically as compared with controls (Table-5).

**Table-5:** Values of haptoglobin and fibrinogen of affected and control calves.

Parameters	Controls n=8	Diseased calves n=42
Haptoglobin g/dl	0.029±0.011	0.045±0.006**
Fibrinogen/s	16.21±5.32	31.23±11.8**

Values are mean±standard error of mean.

** p<0.05

## Discussion

There is no treatment for AKAV infection, and no vaccine is commercially available yet [11,18]. Moreover, prevention of the disease is difficult but could be achieved by ensuring to select resistant breeding females to AKAV infection in non-endemic areas [2]. Akabane disease was detected for the first time in Basrah, Iraq, which might cause economic losses in domesticated ruminants, since dissemble infections of adults, especially dams might lead to further health problems of animals and on months later it will forward to abortion, stillbirths, premature births, and different congenital defects in newborns will follow [19,20].

Diseased calves showed different clinical manifestations which belong to arthrogryposis-hydranencephaly syndrome and also mentioned by others [1,2,4]. As arthrogryposis refers to the fixed flexion of one or more joints and wasting of muscles; however, calves with this condition are commonly called curly calves. Furthermore, severely affected calves are usually born dead and often cause calving difficulties (dystocia), necessitating an embryotomy to deliver them. Furthermore, arthrogryposis usually occurs at an early stage in an outbreak whereas, hydranencephaly occurs when most or all the cerebrum or forebrain is replaced by fluid [10]. It had been documented that if the exposure occurs when the fetus age between 30 and 105 days’ gestation, hydranencephaly is the more common likely outcome. However, if infection occurs between 105 and 150 days’ gestation, arthrogryposis is primarily seen [13]. On the other hand, animals infected earlier in gestation are likely to have multiple limbs affected. Whereas, animals infected later may only have a single affected limb. Since later in gestation the infection occurs, the less severe the arthrogrypotic lesions [18]. Moreover, it is rare for animals show both hydranencephaly and arthrogryposis, but calves infected between days 100 and 120 may exhibit both syndromes [21]. Furthermore, mildly affected calves or lambs may improve their mobility with time. However, most eventually die by 6 months as a result of blindness and other neurological defects [10]. Calves affected as such are often referred to as dummy calves as they are mostly blind, unable to suck, slow, and do not respond to stimuli such as noises. These calves usually appear to be normal at first glance. However, some may have a domed forehead, shortened muzzle, or other abnormalities, such as an undershot or overshot jaw [22]. The clinical cases of arthrogryposis were reflected the complete absence of ventral horn cells in the spinal cord accompanied by neural failure of muscle development. Thus, contracture of the joints results, whereas hydranencephaly manifested by a partial or complete failure of development of the cerebral cortex, the brainstem. However, the cerebellum is usually normal [4,6].

It seems that the main genetic defect or chromosomal elimination occurs in the maternal ancestral. Since naturally occurring recessive genetic defects is common in all species of animals and only become clear if certain breeds of cattle are used very heavily, such that both cows and bulls have common ancestors in their origin, thereby allowing a rare genetic defect to become homozygous in their breeds [23]. On the other hand, the precisely elected a trait bull was also increased the probability of this bull showing up on both sides of many cows lineages, which might result in exhibiting the recessive lethal mutation that terminated with arthrogryposis syndrome [13].

Diagnosis of this disease syndrome could be made on the basis of the clinical signs and can be confirmed by demonstration of antibodies in the blood of calves or dams. Since in the current study, all affected calves show positive seroprevalence results with ELISA test, the same results were also mentioned by Brenner *et al*. [24] and Kittelberger *et al*. [25]. As ELISA system can be adapted for both detection Simbu serogroup viruses in cell capture and the host animals. Furthermore, competitive ELISA (c-ELISA) has been shown highly specific for detection of AKAV antibodies. Moreover, Jun *et al.*, 2012 [7], stated that c-ELISA may be an alternative for increased and sensitive detection of this disease. It had been shown that because of the acute active viral infection, significant increase of lymphocyte number were resulted in the current study in akabane infected calves than in controls [16].

In the current study, ESR was significantly increased in diseased calves than in controls. As it was documented that moderately elevated ESR occurs with anemia and also when inflammatory processes started. However, ESR could also will increase during infection and pregnancy status [16]. Highly ESR values usually had different causes most of them were obvious, such as a severe infection, indicated by an increase in globulins, arthritis and other joint problems, or specific tissue damages (arthrogryposis-hydranencephaly) which were indicated in this study. Since ESR is governable by the equilibrium between factors, play a role before sedimentation, named the fibrinogen factor, and those factors which resisting sedimentation, such as the passive charge of the erythrocytes when an inflammatory process is started. As the high proportion of fibrinogen in the blood causes red blood cells to attach and adhere to each other. Furthermore, the red cells form clumps called rouleaux, which was precipitated more quickly, due to their increased density, as rouleaux formation can also occur in association with some lymphoproliferative conditions when there was a high amount of immunoglobulins secreted [26].

The results of the current study indicated increase acute phase response in diseased calves than controls. Since the acute phase response was considered as a nonspecific response by a single animal to different types of tissue damage. Moreover, the acute-phase response had a large number of physiologic, biochemical, and even nutritional changes involving different organs away from the site(s) of inflammation [27]. The acute phase response is always reflected by specific hepatic proteins calls acute phase proteins which are a group of proteins that change in concentration in animals subjected to new challenges either external or internal such as inflammation, infection, surgical trauma, or even stress [28]. The acute phase proteins consist of negative and positive proteins that show a decrease and an increase in levels, respectively, in response to challenge. Since, positive acute-phase response is regarded as having major functions in opsonization and trapping of microorganisms and their products, in activating the complement system, in binding cellular remnants, in neutralizing enzymes, scavenging free hemoglobin, and radicals, and in modulating the host’s immune response [29,30].

## Conclusions

It has been concluded that in our knowledge, this is the first document established that AKAV infection was diagnosed in calves of Basrah Governorate, Iraq, which will provide useful epidemiological information for cattle and other domesticated animals. Therefore, abortion could be prevented and controlled.

## Authors’ Contributions

KMA was a research coordinator and also drafted the manuscript. HHNA and MAYA were assessed validation method. Both authors read and approved the final manuscript.
